# Virtual Electronic Health Record Technology with Simulation-Based Learning in an Acute Care Pharmacotherapy Course

**DOI:** 10.3390/pharmacy6040123

**Published:** 2018-11-28

**Authors:** James C. Coons, Lawrence Kobulinsky, Deborah Farkas, John Lutz, Amy L. Seybert

**Affiliations:** 1 Department of Pharmacy and Therapeutics, University of Pittsburgh School of Pharmacy, Pittsburgh, PA 15261, USA; lrk26@pitt.edu (L.K.); seyberta@pitt.edu (A.L.S.); 2 Peter M. Winter Institute for Simulation, Education, and Research (WISER), University of Pittsburgh and UPMC, Pittsburgh, PA 15213, USA; farkasd@upmc.edu (D.F.); lutzjw@upmc.edu (J.L.)

**Keywords:** patient simulation, simulation-based learning, virtual electronic health record

## Abstract

Electronic health record (EHR) technology use in the educational setting to advance pharmacy practice skills with patient simulation has not been described previously in the literature. Therefore, the purpose of this study was to evaluate the impact of a virtual EHR on learning efficiency, perceptions of clinical skills, communication, and satisfaction. This was a prospective study conducted in a cardiovascular therapeutics course in the Doctor of Pharmacy curriculum. Students were randomized to use of a virtual EHR with patient simulation or to patient simulation alone (control). The efficiency of learning was assessed by the time to optimal recommendation for each scenario. Surveys (*n* = 12 questions) were administered electronically to evaluate perceptions of clinical skills, communication, and learning satisfaction. Data were analyzed with the Mann–Whitney U or Wilcoxon signed-rank test as appropriate. Use of the virtual EHR decreased the amount of time needed to provide the optimal treatment recommendations by 25% compared to control. The virtual EHR also significantly improved students’ perceptions of their clinical skills, communication, and satisfaction compared to control. The virtual EHR demonstrated value in learning efficiency while providing students with an engaging means of practicing essential pharmacist functions in a simulated setting.

## 1. Introduction

With the rapid adoption of electronic health record (EHR) technology across the continuum of patient care, pharmacists are expected to efficiently collect and evaluate patient’s medication-related data. This information is used to identify medication-related problems and design appropriate therapeutic recommendations. Approximately 70% of pharmacy students report experience in using an EHR, albeit with low levels of confidence when having to perform typical pharmacist duties [1]. Solving medication-related problems and providing cogent pharmacotherapy recommendations, while assuming an appropriate level of confidence and ownership, are expected outcomes of Doctor of Pharmacy (Pharm.D.) student training [2]. The Accreditation Council for Pharmacy Education standards also maintain that graduates be “practice-ready” and “team-ready” [3]. Therefore, opportunities exist to provide pharmacy students with an immersive healthcare training environment to meet these challenges. 

Patient simulation in pharmacy training has been an effective strategy to enhance student pharmacist learning in a large core therapeutics course [4]. However, it is unknown if the integration of a virtual EHR with patient simulation affords a more realistic patient care scenario by providing students with access to clinical information before the encounter. It is also uncertain as to whether student preparedness for pharmacotherapy decision-making is enhanced through the use of a virtual EHR which provides real-time information necessary for data collection and synthesis. Therefore, the purpose of this study was to evaluate the impact of a virtual EHR and patient simulation on learning efficiency and student perception of their learning (clinical skills, communication skills, and satisfaction).

## 2. Materials and Methods 

This prospective study received approval through the University of Pittsburgh Institutional Review Board as exempt research. All 115 students enrolled into the required Pharmacotherapy of Cardiovascular Diseases course (2015-16 academic year) were asked to voluntarily participate in this study. Students that opted in to the study agreed to complete pre- and post-surveys based on their experience with the virtual EHR as well as a clinical pharmacy note which summarized a problem list and treatment recommendations. This course is conducted during the second professional year of the Pharm.D. curriculum and incorporates blended teaching with traditional didactic lectures that precede simulation-based learning using high-technology mannequins. Students complete teamwork activities as a small group during practica assignments which occur at the simulator. After an orientation to the study, student groups were randomized to the intervention or control arm (described below) for each of three disease state-specific practica patient scenarios (acute myocardial infarction complicated by heart failure [MI/HF], acute coronary syndromes [ACS], and dysrhythmias). Intervention and control groups were randomized independently within each patient scenario. There were five to six students in each group. Students remained with their same group as they were assigned to an intervention or control group. The design and structure of each disease state scenario was similar. The allotted time for each scenario per group was approximately ten minutes. 

The provision of clinical information in the virtual EHR (DocuCare^®^, Lippincott Williams and Wilkins) in advance of the simulated encounter was the feature which distinguished the intervention from the control group. The EHR contained a variety of sources of clinical information which mirrored what would be found in a real patient’s record: admission and progress notes, vital signs, baseline laboratory data, home and in-hospital medications, and diagnostic test results. The intervention group had access to the patient’s virtual EHR up to 48 h before their scheduled encounter. Students could then collect, identify, and interpret data from the EHR and begin their assessment of the patient’s condition before the encounter. The control group had the same patient encounter but did not have access to the virtual EHR. Students in the control group were given baseline information (i.e., treatment setting, history of present illness, past medical history, home medications, laboratory parameters, and diagnostics) about the patient at the time of the encounter using supplemental Microsoft PowerPoint^®^ (Microsoft Corporation, Redmond, WA, USA) slides. However, students had to inquire (or were prompted by the facilitator) to gather additional data important for clinical decision-making. All students, regardless of assignment to intervention or control, were provided with the same didactic lecture material before the practicum and were allowed to use notes during the patient encounter. The learning objectives for each case were to: (1) evaluate a new patient with cardiovascular disease and determine the presence of medication-related problems, and (2) integrate patient-specific data using the simulator to develop a pharmacotherapy plan. For each group, the objectives, case content, time in the learning environment, and student-elicited feedback were the same. 

Surveys included a total of 12 questions and were administered electronically before and after the practicum. Survey questions were developed internally by study investigators and are available on request to the corresponding author. Students used a unique identifier so that pre- and post-surveys could be linked, but this was not disclosed to study investigators. All students completed a baseline survey before the first assigned scenario. Each student completed the post-survey twice after serving in the intervention group. However, only the first completed survey was used for the pre/post-intervention comparison to limit potential bias. The pre- and post-surveys self-assessed student perception of three different learning domains: pharmacotherapy knowledge and clinical skills (domain I), attitudes of ownership (domain II), and communication (domain III). The post-survey was the same as the pre-survey but added a domain of learning satisfaction (domain IV). Survey questions were constructed with a Likert scale from 1 to 5 (1 = strongly disagree and 5 = strongly agree). After the patient encounter, students in the intervention group were required to write a clinical pharmacy note summarizing a problem list with recommendations for treatment and monitoring. Students in the control group were not required to complete a note, although, all students had an opportunity to write a note when they participated in the intervention group. Instructors were responsible for providing an evaluation of completeness and accuracy of documentation through qualitative assessment (narrative feedback via DocuCare^®^). 

Each practica session was conducted at the Peter M. Winter Institute for Simulation Education and Research (WISER). Simulation encounters were conducted in medical education theaters that have the appearance and functionality of patient rooms. The patient simulator used was SimMan (a high-technology mannequin manufactured by Laerdal Medical). Each encounter was conducted in groups of five to six students that were briefed on the current condition of the patient. The simulator provided real-time vital signs and vocal responses. This information was then used by the group to arrive at a diagnosis with subsequent recommendations for pharmacologic management. Each practicum session was facilitated by a team of experienced instructors (one instructor per group) that were responsible for the evaluation of an objective structured clinical assessment. The assessments were graded using an objective pharmacotherapy rubric that was previously tested [5]. Components of the rubric that were evaluated include patient introduction, data collection/interpretation, problem list, pharmacotherapy plan, monitoring, and verbal communication. Through this method, both the timing and quality of clinical decision-making (e.g., time to best treatment recommendation) could be determined. The efficiency of learning was ascertained by the time required to provide the most optimal recommendation(s) for each patient scenario as determined by the course instructors. This was conducted at the simulator in real-time. Of note, the pharmacotherapy rubric was only used to evaluate learning efficiency and not clinical skills, communication, or satisfaction (which were captured by the surveys). 

Qualitative data from the surveys were summarized and presented by theme. Pre- and post-survey data were evaluated with the Wilcoxon signed-rank test. Agree and strongly agree responses were aggregated for each domain. Mann–Whitney U test was used to evaluate efficiency of learning (time to best treatment recommendation) between the intervention and control groups for each disease state scenario. Statistical significance was set at an alpha level of 0.05. All statistical analyses were performed using SPSS version 25 (SPSS, Inc., Chicago, IL, USA). Learning satisfaction responses and qualitative feedback from the survey were also summarized. 

## 3. Results

**Figure 1:**
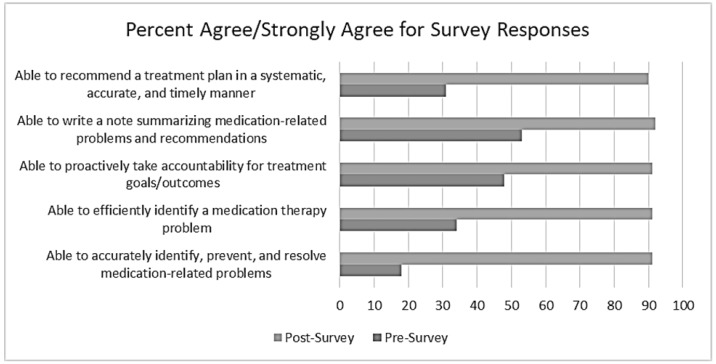
Survey of Clinical and Communication Skills. *p* < 0.001 by Wilcoxon signed-rank test. Survey responses (*n* = 102).

Integration of the virtual EHR decreased the amount of time students needed to provide the best therapeutic recommendations for patients by approximately 25% compared to the control group. The overall median time difference was approximately 2.5 min saved for a typical 10 min patient encounter. However, the impact on each disease state scenario was different. Table 1 shows the efficiency of learning as defined by the time to best treatment recommendation(s) for each scenario. Of note, only the MI/HF case was significantly different between groups. A total of 102 out of 115 s year professional students participated in the study and completed the pre- and post-surveys. Each student served in the intervention and control groups at different time points over the course of the semester. Groups were equally assigned to intervention and control for each practicum. 95% of students agreed or strongly agreed that the use of the EHR contributed positively to their learning and enabled them to efficiently learn new and challenging cardiovascular concepts in the course. A significantly higher proportion of students in the post-survey agreed or strongly agreed that the virtual EHR improved domains related to perceptions of clinical skills, attitudes of ownership, and communication compared to baseline (*p* < 0.001; Figure 1). Finally, annotated qualitative feedback was obtained from survey responses. Student participant responses were consistently positive and described improvements in confidence, problem-solving abilities while adding a sense of realism to the encounter.

## 4. Discussion

The goals of this study were to enhance learning efficiency, communication, and satisfaction by integrating an adaptable and portable virtual EHR with patient simulation. The EHR is a widespread technology adopted as a means of improving the accessibility of patient information as well as the overall safety of healthcare delivery [6]. While most pharmacy students have some exposure to an EHR in clinical practice, few have the confidence and proficiency necessary to effectively use it to carry out core pharmacy functions [1]. In fact, approximately 20% of students are unable to appropriately or completely identify one or more clinical problems and another 15% struggle to define necessary follow-up laboratory monitoring at the completion of a patient visit [7]. Entry-level pharmacy graduates must demonstrate proficiency related to patient-centered care, inclusive of the following: the ability to obtain, interpret, and evaluate patient information, determine the presence of a disease or medical condition, assess the need for treatment or referral, and identify patient-specific factors that impact pharmacotherapy, disease management, and health [2]. Students must also be able to design, implement, monitor, and adjust patient-centered plans and document care activities to facilitate communication and collaboration among the healthcare team [2]. 

The virtual EHR offers an innovative solution to meet the demands of pharmacy graduates by addressing current gaps in the education and training of students in the Pharm.D. curriculum. As a novel instructional technology, the use of virtual EHRs with simulated patients have shown promise toward improving clinical decision-making and documentation skills in pharmacy learners [1,7,8]. While successful in single therapeutics and laboratory-based courses, the adoption of a comprehensive virtual EHR into a large, core therapeutics course to foster critical thinking and problem-solving skills had not been previously evaluated to our knowledge. The required Pharmacotherapy of Cardiovascular Disease course was tested as our first use case for deployment of a virtual EHR. This course was well-suited to test this technology as it encompasses lecture-style delivery of scientific content and therapeutic application, as well as patient simulation for case-based learning. The virtual EHR was embedded into the course to better engage students in an active-learning strategy that emphasized the assimilation of patient data, accountability in clinical and communication skills while providing an efficient mechanism for student evaluation. Additionally, the virtual EHR was linked with patient simulation technology to further enhance learning associated with this teaching method. The traditional means of student evaluation using patient simulation (e.g., objective pharmacotherapy rubric only for the control group) was extended with the use of the virtual EHR in the intervention group by providing a qualitative assessment of pharmacy notes written by the students after the patient encounter. The construct of this course is similar to other Pharm.D. courses. Therefore, the virtual EHR is feasible for rapid adoption throughout the curriculum. Furthermore, the novel teaching methods developed for the effective use of this technology could be scalable to other programs in the health sciences where a virtual EHR would facilitate patient-centered learning. 

Our study was innovative in that it provided students with an engaging and interactive opportunity to practice essential pharmacist functions in a simulated setting as they learn to apply therapeutics concepts without risk to a real patient. The virtual EHR enabled access to clinical information needed to identify and solve medication-related problems, simplify therapeutic regimens, decrease adverse events, and appropriately monitor therapy. Students were also afforded the opportunity to write pharmacist notes and receive prompt and continuous feedback from the instructor through the virtual EHR, which provided a more realistic patient encounter that required higher order thinking and improved documentation skills [7,8]. This educational strategy is supported by data which demonstrated the effectiveness of active-learning techniques which leverage simulated patient cases, Internet-based medical charts, and other electronic records [1,7,8].

The virtual EHR complements patient simulation by integrating a workflow that aligns with realistic patient encounters: (1) review patient information to assess for medication-related problems in advance of the encounter; (2) gather additional clinical information from the patient; (3) provide therapy recommendations, and (4) document a plan with recommendations in the medical record. Use of the virtual EHR also better prepared students for patient encounters at the simulator and increased efficiency of learning (students showed a faster time to appropriate therapy recommendations while minimizing data collection at the patient encounter). Finally, the virtual EHR was well-received by students as evidenced by favorable responses to the survey questions and qualitative feedback which showed improvements in clinical skills, communication, and overall learning satisfaction. Some common themes from the student surveys were that the virtual EHR enabled them to be more confident and prepared for treating patients, and led to a better learning experience. Students also expressed that the patient encounters were more realistic and aligned with what they had experienced during their direct patient experiences.

The efficiency of learning, as measured by the time to the best treatment recommendation, was improved overall in the intervention group. The time savings realized in aggregate led to more effective use of teaching through additional de-briefing with the instructors and student feedback. As clinicians that practice in the cardiovascular setting, an approximate 25% time savings (or mean of 2.5 min) that students achieved would reasonably translate to more timely recommendations for high acuity patients. The time to treatment recommendation difference was evident for both the MI/HF and ACS scenarios in favor of the intervention group, although only the MI/HF case reached statistical significance. It is unclear why no numerical or statistical difference was seen in the dysrhythmias’ scenario, however, it is possible that the management of this disease state was more challenging for students regardless of the technology used. We included a convenience sample of students in a therapeutics class for the study, but the limited size of intervention and control groups overall could have impacted our ability to detect smaller differences in the time to best treatment recommendation between groups. Although multiple instructors were used to evaluate learning efficiency we did make every effort to harmonize the training and structure of the patient simulations between groups. In addition, all students were given some basic introduction and training to the virtual EHR before the study. Students completed two practica which required the use of the virtual EHR. Having access to patient information in the EHR beforehand in the intervention group could have facilitated student learning independent of the EHR itself. Students were given access up to 48 h in advance but could choose to review the information at their discretion. The extent to which these factors impacted the study is uncertain. Nonetheless, students learned to navigate the EHR to find the most relevant information in advance of the patient encounter. 

Regarding the survey instrument, we did not control for the number of virtual EHR experiences that students had before completion of the post-survey in the intervention group. Based on randomization, students could have had up to two experiences before the post-survey was completed. Therefore, it is possible that student perception from their prior experiences could have impacted their survey responses. 

## 5. Conclusions

Integration of a virtual EHR with patient simulation improved learning efficiency, as defined by the time to the most appropriate therapeutic recommendation, compared to patient simulation alone. Furthermore, students’ perceptions of clinical skills, communication, and learning satisfaction were also improved. Our experience with the virtual EHR demonstrated value by providing students with an engaging and interactive means of practicing essential pharmacist functions in a simulated setting. The virtual EHR complements patient simulation by integrating a workflow that aligns with realistic patient encounters. Consequently, future study and expansion of this novel technology into pharmacy curricula should be considered. 

## Figures and Tables

**Table 1:** Efficiency of Learning.

Cohort	Case Scenario	Time to Best Recommendation (Minutes) ^†^	Time Difference to Decision (Minutes)	*p* Value ^‡^
Intervention	MI/HF	5.4 (4.6, 7.6)	2.8	0.048
Control		8.2 (7, 16.3)		
Intervention	ACS	5.5 (4.5, 10.6)	5.1	0.153
Control		10.6 (7.4, 13.3)		
Intervention	Dysrhythmia	7.9 (6, 9.8)	0	1
Control		7.9 (7.1, 9.7)		

^†^ Data expressed as median (interquartile ranges). ^‡^ Mann–Whitney U test. MI/HF = myocardial infarction/heart failure. ACS = acute coronary syndrome.
